# The ABC of Generalized Coordination Numbers and Their Use as a Descriptor in Electrocatalysis

**DOI:** 10.1002/advs.202207644

**Published:** 2023-04-27

**Authors:** Federico Calle‐Vallejo

**Affiliations:** ^1^ Nano‐Bio Spectroscopy Group and European Theoretical Spectroscopy Facility (ETSF) Department of Advanced Materials and Polymers: Physics Chemistry and Technology University of the Basque Country UPV/EHU 20018 Av. Tolosa 72 San Sebastián Spain; ^2^ IKERBASQUE Basque Foundation for Science Plaza de Euskadi 5 Bilbao 48009 Spain

**Keywords:** computational electrocatalysis, coordination numbers, coordination‐activity plots, descriptor‐based analysis, selectivity maps, structural sensitivity

## Abstract

The quest for enhanced electrocatalysts can be boosted by descriptor‐based analyses. Because adsorption energies are the most common descriptors, electrocatalyst design is largely based on brute‐force routines that comb materials databases until an energetic criterion is verified. In this review, it is shown that an alternative is provided by generalized coordination numbers (denoted by $\bar{\mathrm{CN}}$ or GCN), an inexpensive geometric descriptor for strained and unstrained transition metals and some alloys. $\bar{\mathrm{CN}}$ captures trends in adsorption energies on both extended surfaces and nanoparticles and is used to elaborate structure‐sensitive electrocatalytic activity plots and selectivity maps. Importantly, $\bar{\mathrm{CN}}$ outlines the geometric configuration of the active sites, thereby enabling an atom‐by‐atom design, which is not possible using energetic descriptors. Specific examples for various adsorbates (e.g., *OH, *OOH, *CO, and *H), metals (e.g., Pt and Cu), and electrocatalytic reactions (e.g., O_2_ reduction, H_2_ evolution, CO oxidation, and reduction) are presented, and comparisons are made against other descriptors.

## Graphical abstract

In this review, the basics of generalized coordination numbers ($\bar{\mathrm{CN}}$ or GCN) and their use to capture adsorption‐energy trends, elaborate electrocatalytic activity plots, and selectivity maps are shown. Importantly, $\bar{\mathrm{CN}}$ outlines the geometry of the active sites, allowing for atom‐by‐atom catalyst designs. Examples are provided for various adsorbates, metals, and electrocatalytic reactions, along with comparisons against widespread descriptors.

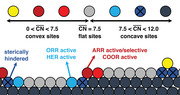

## 1 Introduction

**Figure 1:**
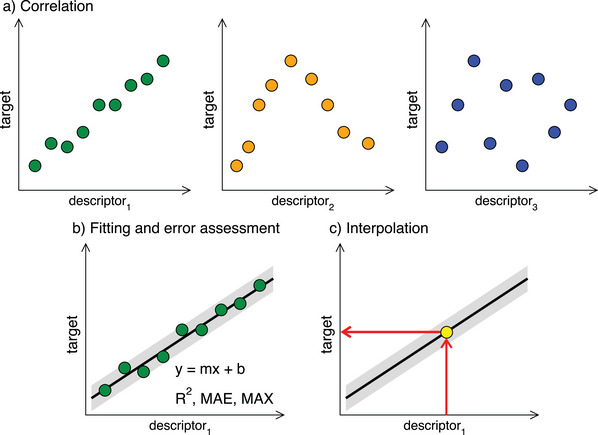
The ABC of descriptor‐based analysis. a) Correlation: the target quantity is plotted as a function of several different descriptors. b) Fitting and error assessment: fits are made, and regression coefficients and mean/maximal absolute errors (MAEs and MAXs) calculated. Based on the performance of the descriptors, an informed choice among different descriptors can be made. The gray band around the fit indicates its accuracy in the description of the data. c) Interpolation: the fit is used to predict with a given accuracy the value of the target that corresponds to a known value of the descriptor. Extrapolations should be avoided.

Nowadays, descriptor‐based analyses are omnipresent in computational catalysis.^[^ ^1^, ^2^, ^3^, ^4^, ^5^ ^]^ I got familiar with the concept during my chemical engineering studies, as the use of dimensionless numbers and combinations thereof as descriptors of transport phenomena is widespread in that field.^[^ ^6^ ^]^ **Figure** **1** schematizes the most basic descriptor‐based analysis one can think of, namely, a 2D plot in which a quantity that is hard to compute or measure, hereon “the target,” is plotted as a function of an easily computable quantity, hereon “the descriptor.” If several descriptors are available, a choice is to be made. High correlation coefficients (*r*) and low mean and/or maximum absolute errors (MAEs or MAXs) are good choices. The simplicity of the fitting function is a good criterion, too, as it is easier to deal with, let us say, a linear function compared to a cubic polynomial.

The ease of calculation of the descriptor is also an important factor, as some descriptors are already tabulated (number of electrons and periodic properties such as electronegativity or ionization potential)^[^ ^7^, ^8^, ^9^ ^]^ or can be readily measured or computed (bond strengths, bulk heat of formation),^[^ ^9^, ^10^, ^11^ ^]^ while others require the making of complicated measurements or electronic‐structure calculations and additional data treatment (work function,^[^ ^7^, ^12^ ^]^ band centers,^[^ ^13^, ^14^ ^]^ integrated crystal orbital overlap population, and crystal orbital Hamilton population^[^ ^7^, ^15^ ^]^). It is important to note that machine learning can be used to obtain regressions with several different descriptors,^[^ ^16^, ^17^, ^18^, ^19^, ^20^ ^]^ and that some descriptors are analytically linked or at least proportional to each other.^[^ ^7^, ^21^ ^]^

An adsorption energy can be used to calculate another, provided that there is some sort of similarity between the two adsorbates. In other words, adsorption energies can both be descriptors and targets. When the adsorption energies of a pair of species 1 and 2 on several different materials are linearly related (i.e., Δ*E*_2_ = *m*Δ*E*_1_ + *b*), a “scaling relation” is said to be formed. The first scaling relations were established between adsorbed atoms and their hydrogenated counterparts (e.g., *OH vs *O; *NH_2_ and *NH vs *N; *CH_3_, *CH_2_, and *CH vs *C)^[^ ^22^ ^]^ but it was later shown that the atoms binding to the surface can be different (e.g., *S vs *O, *P vs *N, and *Si vs *C),^[^ ^23^ ^]^ and that there are as well scaling relations between adsorbed metals and reaction intermediates.^[^ ^24^ ^]^

The slope of scaling relations (*m*) is most often positive and can be calculated on the basis of simple electron‐counting rules,^[^ ^22^, ^23^ ^]^ while the offset (*b*) is generally structure‐sensitive, such that undercoordinated sites have more negative offsets than overcoordinated sites.^[^ ^25^, ^26^, ^27^ ^]^ There are known exceptions to all this: scaling relations exist that have negative slopes,^[^ ^7^ ^]^ the offset is not structure‐sensitive when the slope is unity (*m* = 1),^[^ ^25^, ^26^, ^27^, ^28^ ^]^ covalence can cause significant deviations from the expected value of the slope,^[^ ^29^ ^]^ and scaling relations between two non‐scalable adsorbates can be made by interplaying ligand and coordination effects.^[^ ^30^ ^]^ I would like to stress at this point that scaling relations are a wide and deep topic, and comprehensive reviews on the subject are available in the literature.^[^ ^31^, ^32^, ^33^ ^]^

While scaling relations are energy–energy correlations, there are other types of correlations. The ones I will discuss in this article are majorly structure–energy correlations for transition metals. The descriptor I normally use for that purpose is called “generalized coordination number” ($\bar{\mathrm{CN}}$, often referred to as GCN) which, as the name indicates, is a generalization of the conventional concept of coordination number (cn). In the following, I will first discuss the basics of $\bar{\mathrm{CN}}$, the extensions made to it to incorporate strain and some alloying effects and its applications in computational electrocatalysis. Admittedly, this is by no means an exhaustive literature review on the subject but rather a personal account on the development and use of generalized coordination numbers.

## 2 Discussion

Before proceeding, it is pertinent to mention that the results presented in this section were calculated using VASP, a density functional theory (DFT) code^[^ ^34^ ^]^ that uses plane‐wave basis sets. The exchange‐correlation functional was PBE,^[^ ^35^ ^]^ and the projector augmented‐wave method was used.^[^ ^36^ ^]^ When using DFT, it is advisable to bear in mind that chemical accuracy is 1 kcal mol^−1^ (≈ 0.04 eV) and that the accuracy of DFT at the level of the generalized gradient approximation is around 0.20 eV.^[^ ^37^ ^]^ An acceptable fit, in my opinion, has *r* > 0.85 and MAE < 0.20 eV. A good fit has *r* > 0.90 and MAE < 0.10 eV. Besides, it is desirable that MAE and MAX be as similar as possible to prevent great departures from the fit.

The energetics of proton–electron pairs was calculated using the computational hydrogen electrode, and the overpotentials (*η*) and limiting potentials (*U*_L_) were calculated based on the largest uphill reaction step (for an oxidation reaction: *η* = *U*_L_ − *U* ^0^ = max (Δ*G*_i_)/*e* ^−^ − *U* ^0^, where *U* ^0^ is the equilibrium potential).^[^ ^38^ ^]^ The largest uphill electrochemical step is called potential‐limiting step (PLS). Gas‐phase corrections were applied to ensure a proper description of the equilibrium potentials and the overall reaction energies.^[^ ^39^ ^]^ Adsorbate–solvent interactions were assessed by means of water bilayers and/or micro‐solvation approaches explicitly describing the first solvation shell of the adsorbates.^[^ ^40^, ^41^, ^42^, ^43^ ^]^

Finally, it is worth noting that the analyses shown below presuppose that there are no significant surface reconstructions upon adsorption, such that the geometric and electronic structures of the clean active sites are representative of those with adsorbates. While this is a fair approximation in many cases, there can be exceptions for strong chemisorbates and/or large surface coverage of species.^[^ ^44^ ^]^ Besides, the coupling of phonon modes to vibrational modes of key reaction intermediates is an alternative to lower the activation energy of catalytic reactions without modifying the number of nearest neighbors of the active sites.^[^ ^45^ ^]^

### 2.1 Initial Definition and Extensions

Coordination numbers are probably the simplest geometric descriptor for trends in adsorption energies on pure metal surfaces. For a face‐centered cubic (fcc) metal, they range between 0 and 12, where 0 is a free atom and 12 is an atom in the bulk of a crystalline solid. To say, for instance, that a given metal atom has cn = 9 means that there are nine metal atoms around it located at approximately the interatomic distance seen in the bulk (*d*). The correlation between adsorption energies and coordination numbers for pure metals stems from the fact that a bulk metal atom has all the neighbors it can have and, as a result, is sterically hindered and chemically inactive. When metal‐metal bonds are broken and the metal atom becomes more and more exposed, it is increasingly predisposed to the adsorption of species. In principle, creating metal—adsorbate bonds compensates for the electron density lost upon the decrease of nearest neighbors. In other words, as the coordination number of the metal atom is progressively lowered, it is reasonable to expect that it will tend to bind adsorbates more strongly. This simple but far‐reaching notion is supported by the analytical connection between d‐band centers and coordination numbers explained later in this section.^[^ ^21^ ^]^

It is important to note that the conventional notion of coordination number tacitly assumes that all neighbors are equivalent and identical to those in the bulk. However, at a surface this is not true, as the neighbors have themselves various coordination numbers. The question is then how to account in simple terms for the dissimilarities among the neighbors. Generalized coordination numbers do it by considering the coordination of the first nearest neighbors or, in other words, the second nearest neighbors. This is done in Equation (1) for an atom *i* with *n_i_* first nearest neighbors.^[^ ^21^, ^46^ ^]^

(1) $$\bar{\mathrm{CN}}\left(i\right)=\sum_{j=1}^{n_{i}}\frac{\mathrm{cn}\left(j\right)}{{\mathrm{cn}}_{\max}\;}$$

where cn(*j*) are the coordination numbers of the first nearest neighbors and cn_max_ is the maximal coordination of a given site, as found in the bulk (e.g., cn_max_ = 12 for a single‐atom site in an fcc crystal). It is worth noting that if all neighbors are identical and equivalent to the bulk neighbors, namely if cn(*j*) = cn_max_ , Equation (1) transforms into the conventional definition of coordination numbers in Equation (2).^[^ ^47^ ^]^

(2) $$\bar{\mathrm{CN}}\left(i\right)=\sum_{j=1}^{n_{i}}\frac{{\mathrm{cn}}_{\max\;}}{{\mathrm{cn}}_{\max}\;}=n_{i}=\mathrm{cn}\left(i\right)$$

**Figure 2:**
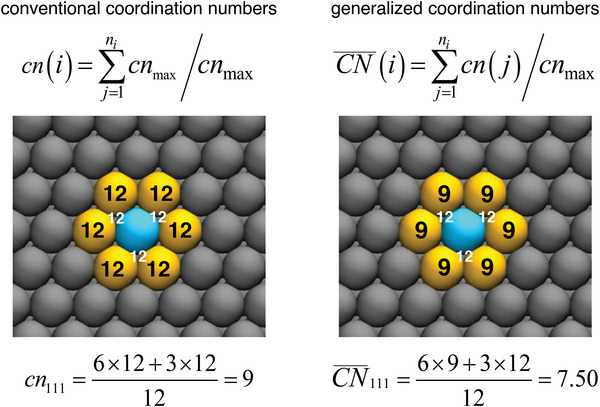
Evaluation of conventional (left) and generalized (right) coordination numbers for a surface atom at a (111) terrace. The atom *i* for which cn and $\bar{\mathrm{CN}}$ are assessed is shown in blue, its first nearest neighbors on the surface layer are shown in yellow, and other surface atoms in gray. The coordination number of the first nearest neighbors is shown in every case. There are three first nearest neighbors in the subsurface layer which cannot be seen from the displayed top view, but their coordination numbers are provided in white.

The use of Equations (1) and (2) is illustrated in **Figure** **2** for a surface atom at a (111) terrace. Because Equations (1) and (2) are arithmetic, $\bar{\mathrm{CN}}$ and cn do not require any electronic‐structure calculations to be assessed and are, hence, inexpensive descriptors. An interesting feature of Equation (1) is that it allows for the evaluation of the $\bar{\mathrm{CN}}$ of multiatom sites, for instance twofold bridge sites and three‐ or four‐fold hollow sites by adjusting the value of cn_max_ (18, 22, and 26 for bridge, three‐ and four‐fold hollow sites, respectively).^[^ ^21^, ^46^ ^]^ This is useful for adsorbates bound to several surface atoms such as *O, *N, and *C. Further examples on the assessment of $\bar{\mathrm{CN}}$ can be found elsewhere.^[^ ^21^, ^46^, ^47^, ^48^ ^]^

Because the concepts of nearest neighbors and conventional coordination numbers also exist on hcp crystals, Equation (1) has been used for metals (Ru, Zn) in that crystalline system.^[^ ^49^, ^50^ ^]^ Equation (1) may as well be used for bcc metals by adjusting cn_max_ (e.g., 12 for top sites on fcc and hcp metals and 8 for bcc metals), but I am not aware of any work in which $\bar{\mathrm{CN}}$ has been used for those.

It is worth adding here that $\bar{\mathrm{CN}}$ is currently defined and used solely for transition metals and some of their alloys. As the bonds in compounds with oxidized metal atoms such as oxides, nitrides, or carbides are more complex than those of metals, to my knowledge there is no straightforward extension of $\bar{\mathrm{CN}}$ in the literature to describe adsorption‐energy trends on those materials. However, recent work on “adjusted coordination numbers” for oxides gives hope for the development of simple coordination‐based descriptors for oxidized metal compounds.^[^ ^51^ ^]^

As alluded before, the connection between $\bar{\mathrm{CN}}$ and adsorption energies to be presented and discussed in the next section is not merely incidental. In fact, generalized coordination numbers are connected analytically to the d‐band centers of transition metals (*ε*_d_) by means of Equation (3),^[^ ^21^ ^]^ and the connection between d‐band centers and adsorption energies is well known.^[^ ^13^ ^]^

(3) $$\varepsilon_{d}^{\mathrm{surf}}\approx \varepsilon_{d}^{\mathrm{bulk}}+\frac{E_{\mathrm{COH}}}{2\theta_{d}f}\left(\frac{{\bar{\mathrm{CN}}}_{\mathrm{surf}}}{{\bar{\mathrm{CN}}}_{\mathrm{bulk}}}-1\right)$$

where the subindices surf and bulk refer to the surface and the bulk of the material, *E*_COH_ is the cohesive energy of the metal, *θ*_d_ is the d‐band occupation, and *f* is a factor that connects the d‐band centers calculated over the entire d‐band and those up to the Fermi level. Equation (3), which is derived from bond‐cutting considerations,^[^ ^52^ ^]^ suggests that the d‐band center of a surface atom is approximately that of a bulk atom plus a correction that is directly proportional to the generalized coordination number of that surface atom. Now, it is known that surface atoms with more negative d‐band centers tend to bind adsorbates more weakly than those with less negative d‐band centers.^[^ ^13^ ^]^ In essence, the geometric and electronic structures of transition metal surfaces are closely connected and determine their adsorption behavior to a great extent.

Strain can also be regarded as a manifestation of generalized coordination. In other words, as much as an atom “feels” more the presence of its neighbors when it has a larger number of second nearest neighbors, having the neighbors closer or farther compared to the bulk equilibrium distances also modifies its generalized coordination. After some mathematical considerations, it is possible to derive a strain‐sensitive generalized coordination number (${\bar{\mathrm{CN}}}^{*}$) that is connected to the regular one through Equation (4).^[^ ^47^ ^]^

(4) $${\bar{\mathrm{CN}}}^{*}\left(i\right)=\frac{1}{1+S}\bar{\mathrm{CN}}\left(i\right)$$

where *S* is the percentage of lattice strain. Note that compressive strain should be treated as a negative number in Equation (4), while tensile strain is positive. For example, if the (111) site in Figure 2 is compressed by 4%, then its generalized coordination number increases from 7.50 to: ${\bar{\mathrm{CN}}}^{*}=7.5/\left(1-0.04\right)=7.81$.

To close this subsection, I would like to annotate that similar^[^ ^53^ ^]^ or more sophisticated coordination‐based descriptors exist.^[^ ^16^, ^54^, ^55^ ^]^ While all of the examples shown in this review are for pure transition metals, supervised machine learning has recently been used to modify generalized coordination numbers such that they can be applied on Pt_3_M (M = Co, Ni, Cu) alloy nanoparticles.^[^ ^56^ ^]^ Besides, ${\bar{\mathrm{CN}}}^{*}$ was recently enabled to incorporate some alloying effects, which helps elucidate the location of active sites at alloys and opens the way for new applications in electrocatalysis.^[^ ^57^ ^]^

### 2.2 Trends in Adsorption Energies

**Figure 3:**
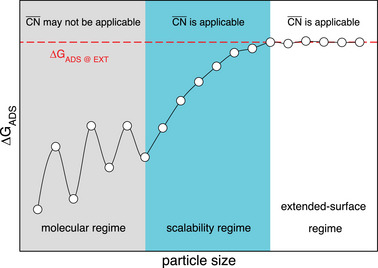
Schematics of the trends in adsorption energies as a function of particle size. Three zones are observed: at low particle sizes, there is a molecular regime where small size and shape changes induce drastic changes in the adsorption energies. At intermediate sizes there is a scalability regime in which finite‐size effects disappear progressively as the particles get larger. For large particles and extended surfaces there is an extended‐surface regime where there are no finite‐size effects.

So far, I have formally defined $\bar{\mathrm{CN}}$ without testing it as a descriptor. In this subsection, I will illustrate its performance for capturing trends in adsorption energies and show some known cases in which it performs better than cn and *ε*_d_. I must start by saying that the notion that coordination numbers and surface properties covary is well documented for metals.^[^ ^13^, ^58^, ^59^ ^]^ In fact, conventional coordination numbers have been used as descriptors for adsorption energies by various authors.^[^ ^60^, ^61^, ^62^, ^63^, ^64^ ^]^ Nevertheless, the performance of cn is unsatisfactory when finite‐size effects^[^ ^65^ ^]^ are present at the adsorption sites, which happens on small and medium nanoparticles,^[^ ^60^, ^66^, ^67^ ^]^ as outlined in **Figure** **3**. In principle, cn is only applicable in the extended‐surface regime schematized in the figure and probably at the beginning of the scalability regime, namely for middle‐sized and large nanoparticles and extended surfaces, for which finite‐size effects are moderate. By considering second nearest neighbors, $\bar{\mathrm{CN}}$ allows for the inspection of the trends in the scalability and extended‐surface regimes.

**Figure 4:**
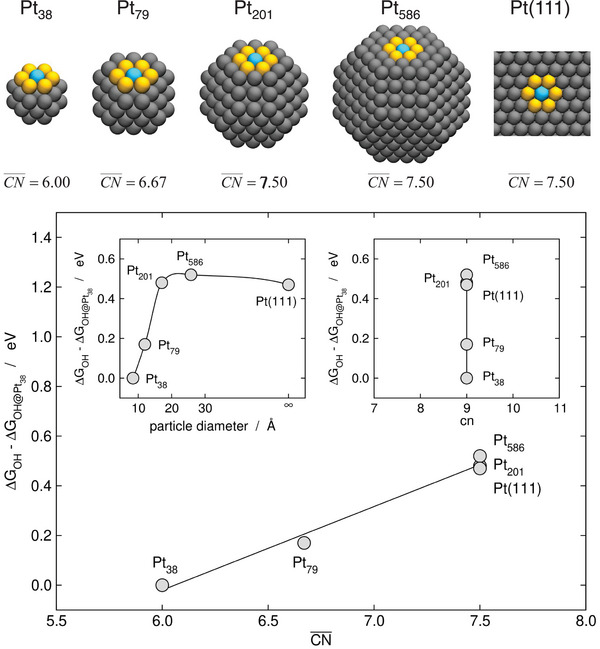
Illustration of finite‐size effects on Pt nanoparticles. Top: sites with cn = 9 on Pt nanoparticles with 38, 79, 201, and 586 atoms, and Pt(111). The values of $\bar{\mathrm{CN}}$ are provided in each case. The color code is the same as in Figure 2. Bottom: trends in atop *OH adsorption energies using Pt_38_ as a reference for the sites shown in the top panel. The scalability and extended‐surface regimes are shown in the left inset, the limitations of cn to distinguish the sites are shown in the right inset, and the nearly linear description offered by $\bar{\mathrm{CN}}$ is shown in the main panel.^[^ ^21^, ^68^, ^69^ ^]^ Upper part: Reproduced with permission.^[^ ^68^ ^]^ Copyright 2019, Royal Society of Chemistry.

The range of applicability of $\bar{\mathrm{CN}}$ and its advantages with respect to cn are illustrated in **Figure** **4** for *OH adsorption on Pt nanoparticles. The top panel of Figure 4 shows five sites with cn = 9 on increasingly large nanoparticles (Pt_38_, Pt_79_, Pt_201_, Pt_586_) and Pt(111). All blue sites in Figure 4 have nine neighbors but the neighbors have dissimilar coordination numbers. As a result, $\bar{\mathrm{CN}}$ is in the range of 6.00–7.50. The bottom panel of Figure 4 shows that the *OH adsorption energies change by as much as ≈0.5 eV between Pt_38_ and Pt(111). Besides, Pt_38_ and Pt_79_ are in the scalability regime, Pt_201_ is likely at the boundary between the scalability and extended‐surface regimes, whereas Pt_586_ and Pt(111) belong to the extended‐surface regime. Interestingly, while cn is unable to capture the trends in adsorption energies, $\bar{\mathrm{CN}}$ provides a nearly linear correlation that fits well all of the points (Figure 4 bottom, right inset and main panel, respectively).

I emphasize, however, that $\bar{\mathrm{CN}}$ is probably not useful in the molecular regime (presumably below Pt_38_ in this case) because materials properties in that range of sizes may change dramatically by adding or removing a single atom. This means that the trends are in general not monotonic in that regime, which causes the failure of conventional descriptors and calls for more advanced ones.

**Figure 5:**
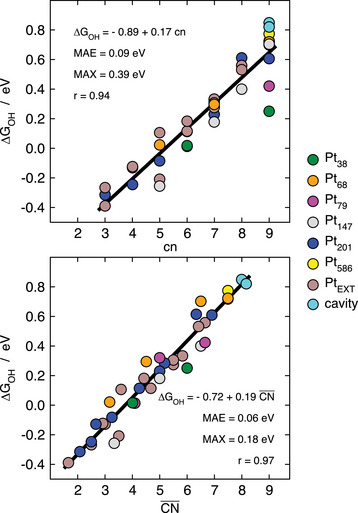
Trends in atop *OH adsorption energies for sites at nanoparticles and extended surfaces of Pt. The category “cavity” refers to small holes created on Pt(111) to increase $\bar{\mathrm{CN}}$ without changing cn. Reproduced with permission.^[^ ^69^ ^]^ Copyright 2015, AAAS.

Data for atop *OH adsorption at numerous active sites of nanoparticles and extended surfaces are provided in **Figure** **5**, where the linear regressions, correlation coefficients, and MAEs show a better performance of $\bar{\mathrm{CN}}$ compared to cn. Moreover, Figure 5 shows that finite‐size effects are more pronounced for certain coordination numbers than others. In particular, they can be as large as 0.6 eV for cn = 9. In addition, the MAX is considerably lower for $\bar{\mathrm{CN}}$ compared to cn: 0.18 versus 0.39 eV.

**Figure 6:**
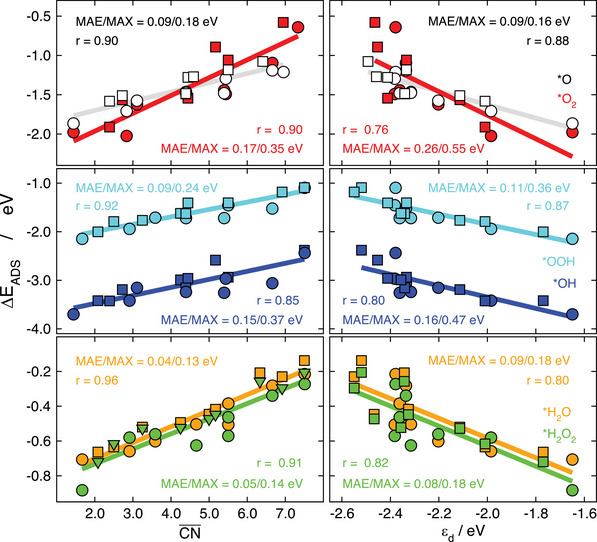
Adsorption energies of *O and *O_2_ (top), *OH and *OOH (middle), and *H_2_O and *H_2_O_2_ (bottom) on extended Pt surfaces (circles) and Pt_201_ (squares). The trends are described using $\bar{\mathrm{CN}}$ (left) and *ε*_d_ (right). The correlation coefficient, MAE and MAX of the linear fits are provided in each case. Reproduced with permission.^[^ ^21^ ^]^ Copyright 2014, Wiley‐VCH.

This is also systematically observed when comparing $\bar{\mathrm{CN}}$ and *ε*_d_, as illustrated in **Figure** **6** for the adsorption energies of *O, *O_2_, *OH, *OOH, *H_2_O, and *H_2_O_2_ on extended surfaces of Pt and on Pt_201_. In nearly all cases the MAXs are visibly lower for the linear fits based on $\bar{\mathrm{CN}}$, and the correlation coefficients are closer to 1, which attests to a higher predictive power of $\bar{\mathrm{CN}}$. The practical reason for the higher accuracy of $\bar{\mathrm{CN}}$ compared to *ε*_d_ on Pt is visible in the right panels of Figure 6. Essentially, the scattering is large in the approximate range of *ε*_d_ from −2.3 to −2.5 eV.^[^ ^21^ ^]^

**Figure 7:**
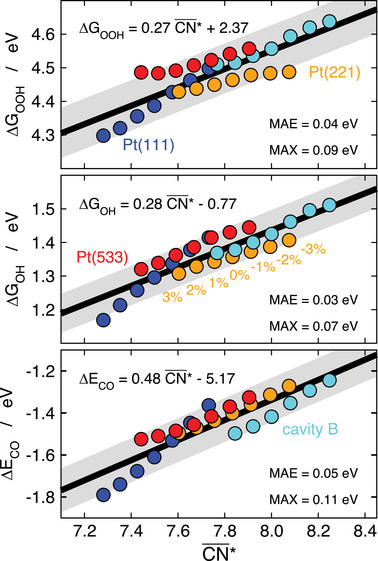
Trends in adsorption energies of *OOH (top), *OH (middle), and *CO (bottom) on sites with cn = 9 at Pt(111), Pt(221), Pt(533), and a small cavity on Pt(111), as a function of strain‐sensitive ${\bar{\mathrm{CN}}}^{*}$. The equation, MAE and MAX of the linear fits are given in each case. Reproduced with permission.^[^ ^47^ ^]^ Copyright 2018, Wiley‐VCH.

Furthermore, **Figure** **7** shows that ${\bar{\mathrm{CN}}}^{*}$ is able to capture the trends in adsorption energies of *OOH, *OH, and *CO on various strained and unstrained Pt sites with low MAEs and MAXs. Interestingly, it has been shown that the errors are even lower if the trends are analyzed separately for each surface site subject to different degrees of strain.^[^ ^47^ ^]^

To close this subsection, I would like to stress that the examples in Figures 4, 5, 6, 7 are all for Pt and oxygen‐bound adsorbates and *CO. However, generalized coordination numbers can and have been applied on other metals and for the adsorption of various species. For instances, carbon‐, nitrogen‐, and oxygen‐containing adsorbates on Au;^[^ ^47^, ^69^, ^70^, ^71^ ^]^ *H^[^ ^48^ ^]^ and *CO^[^ ^72^ ^]^ on Pt; *B, *C, and *CO on Ru;^[^ ^49^ ^]^ oxygen‐ and carbon‐containing intermediates on Cu^[^ ^73^, ^74^, ^75^, ^76^ ^]^ and Co;^[^ ^77^ ^]^ carbon‐bound species on Zn;^[^ ^50^ ^]^ and *H_2_O on Pd.^[^ ^78^ ^]^

### 2.3 Solvation Energies

Water is the most widely used solvent and a vast number of electrocatalytic reactions are either hydrogenations or dehydrogenations (e.g., O_2_ reduction and evolution, CO_2_ reduction and CO oxidation, and nitrate or NO reduction, just to name a few), in which adsorbed intermediates able to make hydrogen bonds with water are recurrently formed. Hence, prior to exposing and analyzing the activity plots based on generalized coordination numbers for some of those reactions, I will spend a few lines in this subsection discussing adsorbate–water interactions, which are often referred to as adsorbate solvation.

In spite of their affordability and advantages for the modeling of (electrified) interfaces,^[^ ^79^, ^80^, ^81^ ^]^ implicit solvent methods have well‐known limitations for the description of solvent–adsorbate hydrogen bonds,^[^ ^82^, ^83^, ^84^, ^85^ ^]^ which may have serious repercussions for the predictiveness of computational catalyst design routines.^[^ ^42^ ^]^ Of course, ab initio molecular dynamics simulations can be carried out,^[^ ^85^, ^86^, ^87^, ^88^ ^]^ but their computational cost is still high and can be prohibitive for nanoparticles with several hundred atoms.^[^ ^89^ ^]^ Alternatively, ice‐like (hexagonal) water bilayers over metal slabs are frequently used to represent metal–water interfaces.^[^ ^38^, ^40^, ^87^, ^90^, ^91^ ^]^ Even more computationally affordable and in agreement with the results of water bilayers is the use of the first solvation shell of the adsorbates to assess their solvation energies, denoted by Ω_ADS_.

**Figure 8:**
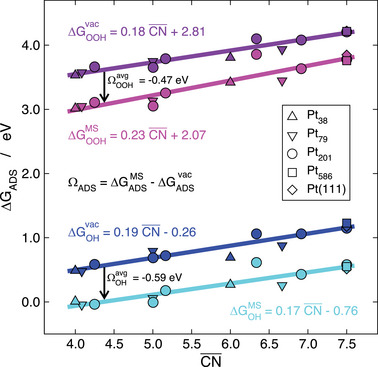
Trends in the adsorption energies of *OH and *OOH on Pt(111) and various Pt nanoparticles as a function of $\bar{\mathrm{CN}}$. The trends in vacuum ($\Delta G_{\mathrm{ADS}}^{\mathrm{vac}}$) and in a micro‐solvated environment ($\Delta G_{\mathrm{ADS}}^{\mathrm{MS}}$) are described by linear fits with similar slopes, such that average solvation corrections of −0.59 ± 0.14 and −0.47 ± 0.13 eV are extracted for *OH and *OOH, respectively. Reproduced with permission.^[^ ^41^ ^]^ Copyright 2019, American Chemical Society.

This is shown in **Figure** **8** for *OH and *OOH adsorption on Pt(111) and Pt nanoparticles of various sizes.^[^ ^41^ ^]^ For these two adsorbates, the first solvation shell consists of three water molecules: two of them donate a hydrogen bond via one of their —H moieties and the other one receives it via their —O moiety. Figure 8 shows that the trends in adsorption energies in vacuum ($\Delta G_{\mathrm{ADS}}^{\mathrm{vac}}$) and in the micro‐solvated environment containing three interfacial water molecules ($\Delta G_{\mathrm{ADS}}^{\mathrm{MS}}$) are well described in both cases by $\bar{\mathrm{CN}}$. Because the lines are nearly parallel, it is possible to say that at low adsorbate coverage the solvation energies are nearly constant and equal to −0.59 ± 0.14 and −0.47 ± 0.13 eV for *OH and *OOH, respectively. I would like to annotate that this simple micro‐solvation approach was recently extended to Ir, Pd, Ag, and Au,^[^ ^43^ ^]^ and can be used in combination with implicit solvent methods and for the assessment of the solvation energies of any adsorbate, not just those able to fit within a symmetric water bilayer.^[^ ^42^, ^75^ ^]^

### 2.4 Coordination‐Activity Plots

The Sabatier principle, which originated from Paul Sabatier's works on hydrogenation catalysis,^[^ ^92^ ^]^ is a widespread, simple, and powerful guideline for the design of catalysts.^[^ ^2^, ^3^, ^5^ ^]^ It states that the best catalyst for a given reaction is one that binds the reactants, intermediates, and products neither too strongly nor too weakly. Strong binding may lead to site blocking and eventual deactivation of the catalyst, whereas weak binding prevents adsorption events and may render the catalytic surface inert. I like to think of the Sabatier principle as a chemical version of Aristotle's golden mean, which says that virtue is found in the middle between two extremes, one of excess and other of deficiency. All in all, the Sabatier principle is merely qualitative, and its quantification is arduous both in experimental and computational terms. Since the maximal catalytic activity is found in the middle between two extremes of low activity, the term “volcano plot,” allegedly coined by Balandin,^[^ ^93^ ^]^ is vastly used for the activity versus descriptor plots in the catalysis literature^[^ ^2^, ^3^, ^5^, ^94^, ^95^ ^]^ that quantify the Sabatier principle.

Before continuing, I take this opportunity to refer the reader to the nuances and criticisms of the Sabatier principle and particularly of volcano plots.^[^ ^96^, ^97^, ^98^, ^99^, ^100^, ^101^ ^]^ It is also worth noting that some authors have reported on the need to quantify the uncertainty of volcano plots, correct their implicit errors, and find the most suitable descriptors for each reaction.^[^ ^102^, ^103^, ^104^ ^]^

Because of the extensive use of adsorption energies as descriptors, most of the afore‐cited quantifications of the Sabatier principle are energetic. This means that the binding energies of optimal catalysts can be calculated but connecting those to structural guidelines is not univocal. In other words, a given binding energy can be displayed by a plethora of active sites, but a given active site displays only one adsorption energy. In my opinion, this has turned catalyst design into a brute‐force screening task in which a considerable number of materials is evaluated until at least one satisfies the search criteria. More than design, I would refer to that practice as materials selection, because the outcome of the screening process is a catalyst that had to be included in the materials database beforehand. Essentially, it is not possible to find an unknown material from a materials’ screening performed over a database of known compounds.

For instance, volcano plots showed that, in terms of *OH adsorption energies, Pt(111) is 0.10–0.15 eV to the left of the volcano plot for the oxygen reduction reaction (ORR, in acid: O_2_ + 4H^+^ + 4e^−^ → 2H_2_O). This concise observation provided a framework to predict or rationalize the high ORR activity of numerous Pt‐based electrodes which indeed bind *OH more weakly than Pt(111).^[^ ^105^, ^106^, ^107^, ^108^, ^109^ ^]^ Although the weakening of *OH adsorption with respect to Pt(111) by 0.10–0.15 eV is certainly a quantitative guideline, in practice it is difficult to anticipate whether a given material will bind *OH more weakly or strongly than another one without making DFT calculations or experimental measurements.

**Figure 9:**
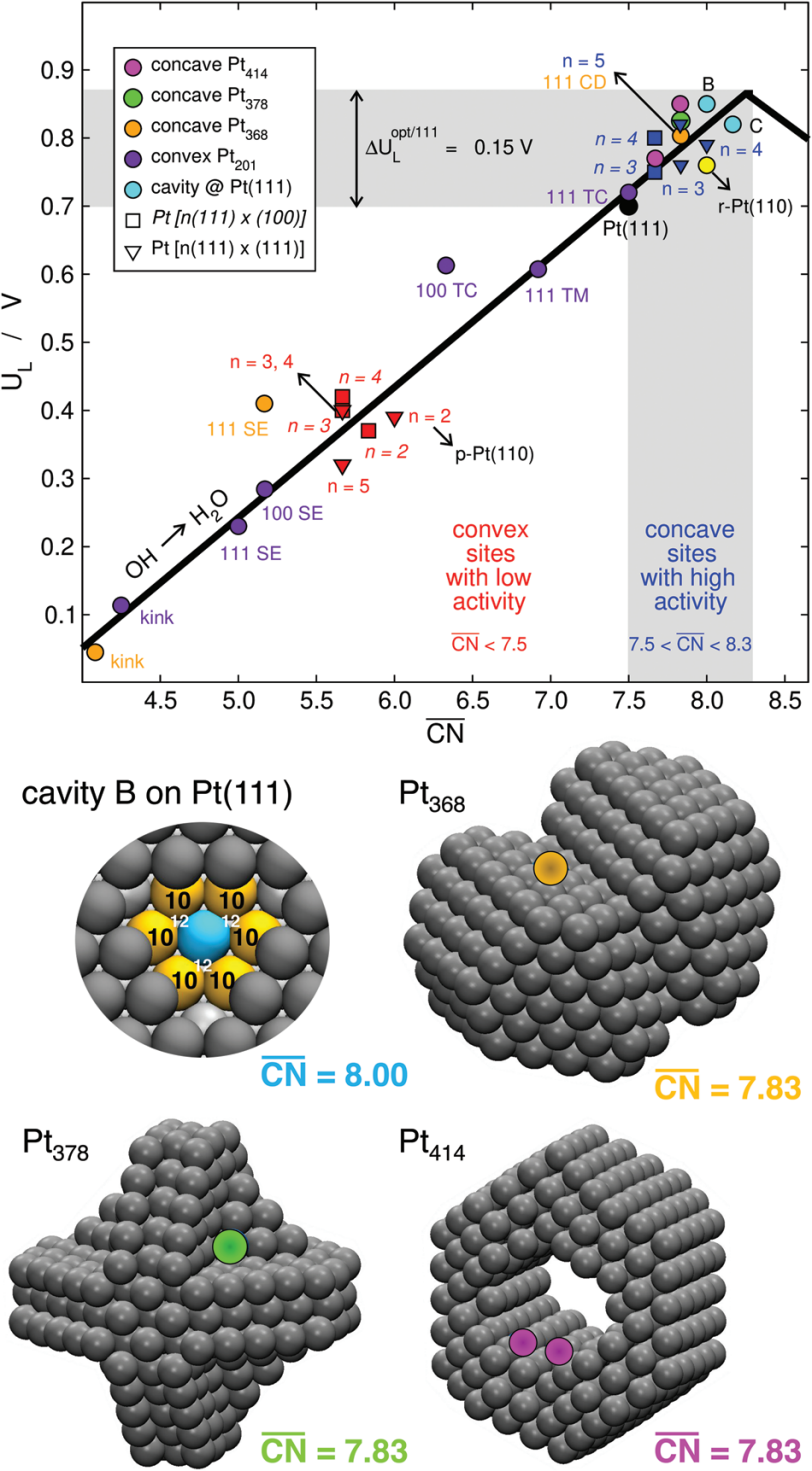
Structural sensitivity of the ORR on Pt sites at extended surfaces and nanoparticles. Top: coordination‐activity plot. Two regions are marked: convex sites with low ORR activity ($\bar{\mathrm{CN}}<7.5$), and concave sites with high ORR activity ($7.5<\bar{\mathrm{CN}}<8.3$). Regular nanoparticle sites and undercoordinated defects are all located in the convex region. Step bottom sites are located in the concave (gray) region. SE: step edge, TC: terrace center, TM: terrace middle, CD: concave defect, *n*: terrace length of stepped surfaces, p‐ and r‐Pt(110): pristine and missing‐row reconstruction of Pt(110).^[^ ^68^, ^69^, ^110^ ^]^ Bottom: cavity B and three concave nanoparticles with highly active sites for the ORR. Cavity B: Reproduced with permission.^[^ ^69^ ^]^ Copyright 2015, AAAS. Pt_368_, Pt_378_, Pt_414_: Reproduced with permisssion.^[110]^ Copyright 2017. Royal Society of Chemistry.

An alternative is the “coordination‐activity plot” in **Figure** **9** (top panel), which describes the trends in limiting ORR potentials among Pt sites in terms of $\bar{\mathrm{CN}}$.^[^ ^69^, ^110^ ^]^ The plot shows that it is possible to enhance the ORR activity of Pt(111) by increasing its $\bar{\mathrm{CN}}$ from 7.5 up to 8.3 (note that subsequent works refined this range to 7.5–8.0 by zooming in on the apex area^[^ ^47^, ^57^, ^111^ ^]^). A first choice to increase $\bar{\mathrm{CN}}$ is the addition of a new nearest neighbor, such that cn increases from 9 to 10. The problem is that sites with cn = 10 have exceedingly large generalized coordination numbers and are sterically hindered. For example, the step bottom of a Pt(211) surface has cn = 10 and $\bar{\mathrm{CN}}=\frac{1}{12}\left(1\;\times \;7+2\;\times \;9+2\;\times \;10+5\;\times \;12\right)=8.75$. As a result, adsorbates on top of it either bind too weakly or diffuse to vicinal sites offering stronger adsorption energies.

If $\bar{\mathrm{CN}}$ is to be increased but adding first nearest neighbors is not an option, second nearest neighbors should be added instead. This is why cavities B and C in Figure 9 have cn = 9 as Pt(111), but their number of second nearest neighbors is different. For example, for cavity B we have: $\bar{\mathrm{CN}}=\frac{1}{12}\left(6\;\times \;10+3\;\times \;12\right)=8.00$. In addition, cn = 9 on the terraces near step bottoms, but the number of second nearest neighbors increases. Conversely, undercoordinated step edges are inactive in view of their low number of first and second nearest neighbors (cn = 7 and $\bar{\mathrm{CN}}<6.0$).

These findings help explain why stepped Pt(111) surfaces are found to be highly active for the ORR^[^ ^110^ ^]^ and motivated the experimental creation of small cavities on Pt(111) by means of three different methods, namely dealloying of a surface alloy, galvanic displacement, and “electrochemical destruction,” and the synthesized catalysts displayed ORR activities up to 3.5 times larger than Pt(111).^[^ ^69^ ^]^ Interestingly, cathodic corrosion has also been used to create cavities with high ORR activities on Pt(111).^[^ ^112^ ^]^ An option for the creation of cavities within Pt nanoparticles is by seizing the nanoscale Kirkendall effect, in which a binary alloy nanoparticle ends up hollow as a result of the different diffusion rates of its components.^[^ ^113^ ^]^

In this order of ideas, knowing that highly active sites on Pt for the ORR have $7.5<\bar{\mathrm{CN}}<8.0-8.3$, it is possible to design active sites from scratch in an atom‐by‐atom fashion without any need for brute‐force screening. Indeed, starting from a large nanoparticle and carving atoms out, the three concave nanoparticles (Pt_368_, Pt_378_, Pt_414_) in the bottom panel of Figure 9 were built, and DFT calculations confirmed the predictions (Figure 9, top panel).^[^ ^110^ ^]^ Of course, the procedure can and has been automatized by various authors,^[^ ^114^, ^115^ ^]^ and the high ORR activity of concave Pt‐based nanoparticles has repeatedly been observed in experiments.^[^ ^113^, ^116^, ^117^ ^]^

Note in passing that the design principle related to the weakening of *OH binding derived from conventional volcano plots is reproduced by coordination‐activity plots ($\Delta U_{L}^{\mathrm{opt}/111}$ in Figure 9). This suggests that coordination‐activity plots conform to volcano plots and, in addition, are able to outline the geometric structure of the active sites.

**Figure 10:**
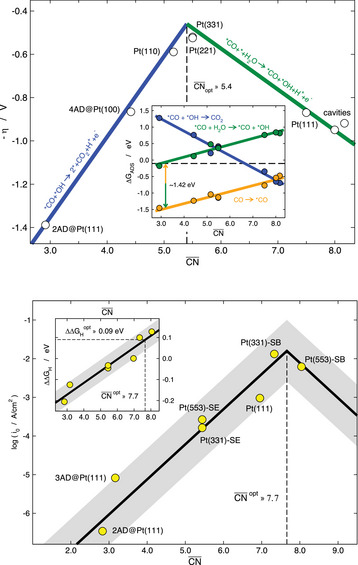
Coordination‐activity plots for two fundamental electrocatalytic reactions catalyzed by Pt electrodes. Top: additive inverse of the CO oxidation overpotential as a function of $\bar{\mathrm{CN}}$. Inset: energetics of the elementary steps. Bottom: exchange current density of hydrogen evolution calculated via microkinetic modelling^[^ ^119^ ^]^ as a function of $\bar{\mathrm{CN}}$. Inset: trends in *H adsorption energies. The optimal value of $\bar{\mathrm{CN}}$ is 5.4 for CO oxidation and 7.7 for hydrogen evolution, so that the former is enhanced by undercoordinated convex sites while the latter is enhanced by overcoordinated concave sites. SE: step edge, SB: step bottom. Reproduced with permission.^[^ ^48^ ^]^ Copyright 2017, American Chemical Society. Reproduced with permission.^[^ ^72^ ^]^ Copyright 2017, American Chemical Society.

Furthermore, **Figure** **10** illustrates the structural sensitivity of the CO oxidation reaction (COOR: CO + H_2_O → CO_2_ + 2H^+^ + 2e^−^)^[^ ^72^ ^]^ and the hydrogen evolution reaction (HER: 2H^+^ + 2e^−^ → H_2_)^[^ ^48^ ^]^ in acid using Pt electrodes. According to the coordination‐activity plot, CO oxidation is enhanced by undercoordinated sites, and the highest activity should be observed for $\bar{\mathrm{CN}}\approx 5.4$, which is slightly lower than the generalized coordination number of a typical step edge, for instance for the step edge of Pt(221): $\bar{\mathrm{CN}}=\frac{1}{12}\left(2\;\times \;7+2\;\times \;9+2\;\times \;11+1\times 12\right)=5.50$. CO stripping voltammetry experiments confirmed this prediction and showed that the onset of the reaction is earlier for stepped surfaces with shorter terraces compared to those with longer terraces (e.g., Pt(331) vs Pt(775)),^[^ ^72^ ^]^ in agreement with previous works.^[^ ^118^ ^]^ Conversely, the HER is predicted to be enhanced by overcoordinated Pt sites at step bottoms ($\bar{\mathrm{CN}}\approx 7.7$). Although this value is similar to what was observed for the ORR, the adsorption sites of the key reaction intermediates are different: top sites for *OH and hollow sites for *H. Again, electrochemical experiments were able to corroborate this prediction and showed that the activity of Pt(111) grows as overcoordinated defects are introduced to it.^[^ ^48^ ^]^

**Figure 11:**
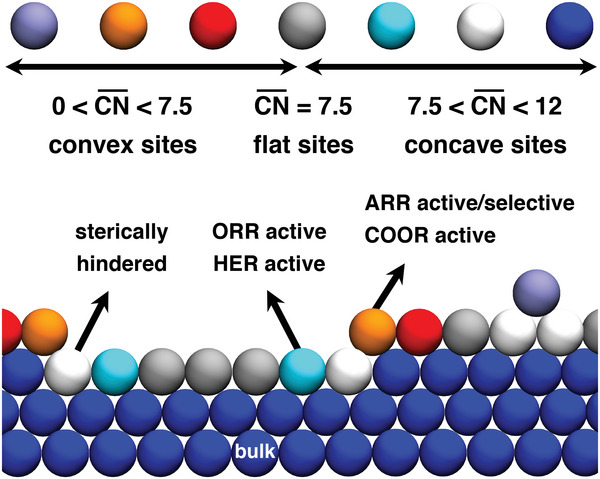
Classification of sites on Pt electrocatalysts according to their generalized coordination number as convex, flat, and concave. In each case, the reactions enhanced by those sites are given. ARR: acetone reduction reaction. ORR: oxygen reduction reaction. HER: hydrogen evolution reaction. COOR: CO oxidation reaction.

As shown in **Figure** **11**, the analysis of the ORR, COOR and HER, enables a classification of Pt sites as either convex ($\bar{\mathrm{CN}}<7.5$), flat ($\bar{\mathrm{CN}}=7.5$), or concave ($\bar{\mathrm{CN}}>7.5$).^[^ ^110^ ^]^ While flat sites typically display fair activities for all three reactions, convex sites enhance the COOR, and concave sites enhance the HER and ORR. This is because Pt(111) is a weak‐binding facet for COOR, such that lowering the conventional and generalized coordination numbers increases its activity, whereas it is a strong‐binding facet for the HER and ORR, such that a higher generalized coordination is beneficial. In the next section, I will show that this type of classification can also be used for selectivity purposes.

A closer look at Figures 9 and 10 makes apparent an interesting fact: *OH is usually a key intermediate on Pt(111), as COOR is limited by a step in which *OH is formed (*CO + * + H_2_O → *CO + *OH + H^+^ + e^−^) and the ORR is limited by one in which *OH is consumed (*OH + H^+^ + e^−^ → * + H_2_O). Hence, in the former case, undercoordination facilitates *OH formation at lower overpotentials, whereas in the latter case overcoordination facilitates *OH hydrogenation also at lower overpotentials.

Before closing this subsection, I would like to make four remarks. First, coordination‐activity plots have also been made for CO_2_ reduction and akin reactions on Cu^[^ ^73^, ^74^, ^76^ ^]^ and Zn,^[^ ^50^ ^]^ and for the ORR and N_2_ reduction on Au.^[^ ^69^, ^71^ ^]^ Second, generalized coordination numbers and the distinction between convex and concave sites have been used to elaborate advanced models of catalytic^[^ ^77^, ^120^, ^121^ ^]^ and electrocatalytic reactions.^[^ ^114^, ^115^, ^122^, ^123^, ^124^, ^125^ ^]^ Third, recently, coordination‐activity plots have also been made that include strain and alloying effects for Pt‐based ORR electrocatalysts and their results are not only in agreement with experiments but also help elucidate the structure and composition of the active sites.^[^ ^47^, ^57^, ^111^ ^]^ Fourth, $\bar{\mathrm{CN}}$ could, in principle, be applied to capture trends for any reaction that displays structural sensitivity. For instance, in reactions within the nitrogen cycle, as cn has been used in the past for the modelling of those with certain success.^[^ ^63^ ^]^

### 2.5 Selectivity Maps

As shown in Figures 9 and 10, activity plots are usually made for a single electrochemical reaction. However, it is common in electrochemistry to have competing reactions and/or competing products within a given reaction. If the products and/or reactions are site‐dependent, one can resort to “selectivity maps” based on $\bar{\mathrm{CN}}$. The underlying principle of those maps is the same as that of coordination‐activity plots: the thermodynamic requirements for opening catalytic pathways vary as a function of $\bar{\mathrm{CN}}$ because the adsorption energies of intermediates are also a function of $\bar{\mathrm{CN}}$. The maps result from overlapping several different coordination‐activity plots and choosing the most favorable one, namely, the one requiring the least overpotential.

Let us consider the case of the acetone reduction reaction (ARR), which displays a peculiar structural sensitivity on platinum electrodes:^[^ ^126^ ^]^ Pt(111) and Pt(100) are not able to reduce acetone and the only electrocatalytic product observed is hydrogen. Moreover, Pt electrodes with (111) terraces and (110) steps (e.g., Pt(110) and Pt(553)) reduce acetone to 2‐propanol via a two‐electron reaction (CH_3_COCH_3_ + 2H^+^ + 2e^−^ → CH_3_CHOHCH_3_), whereas Pt electrodes with (100) terraces and (110) steps (e.g., Pt(510)) reduce acetone to propane via a four‐electron reaction (CH_3_COCH_3_ + 4H^+^ + 4e^−^ → CH_3_CH_2_CH_3_ + H_2_O).

**Figure 12:**
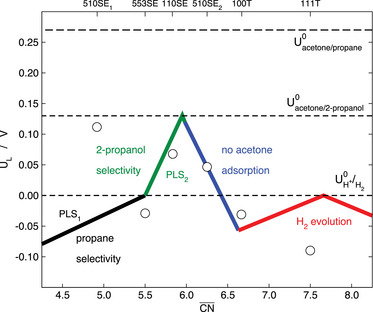
Selectivity map for acetone reduction to 2‐propanol and propane on Pt electrodes. There is only hydrogen evolution for $\bar{\mathrm{CN}}>6.6$; when $6.0<\bar{\mathrm{CN}}<6.6$ the acetone adsorption energy is not strong enough for the reaction to proceed; for $5.5<\bar{\mathrm{CN}}<6.0$ acetone reduction is inclined toward 2‐propanol, whereas for $\bar{\mathrm{CN}}<5.5$ acetone reduction is selective toward propane. The dashed lines correspond to the equilibrium potentials of the competing reactions. SE: step edge, T: terrace. PLS_1_: *CH_3_CCH_3_ + H^+^ + e^−^ → *CH_3_CHCH_3_. PLS_2_: *CH_3_COHCH_3_ + H^+^ + e^−^ → CH_3_CHOHCH_3_. Reproduced with permission.^[^ ^126^ ^]^ Copyright 2019, Springer Nature.

To rationalize this intricate selectivity, one can resort to the selectivity map in **Figure** **12**,^[^ ^126^ ^]^ which correlates the limiting potentials of the HER and those of acetone reduction to 2‐propanol and propane with the generalized coordination numbers of various Pt surface sites. The selectivity map shows that there is only H_2_ evolution for $\bar{\mathrm{CN}}>6.6$. In the $\bar{\mathrm{CN}}$ range of 6.0–6.6, the ARR to 2‐propanol is more favorable than the HER but acetone adsorption is still endergonic. When $\bar{\mathrm{CN}}$ is between 5.5 and 6.0, the conditions are favorable for the selective production of 2‐propanol, whereas for $\bar{\mathrm{CN}}<5.5$ the ARR becomes selective toward propane. In broad terms, Figure 12 suggests that before advanced kinetics‐based analyses are made to explain the selectivity of complicated reaction networks, a more affordable thermodynamic analysis using a structure‐sensitive descriptor might suffice.

**Figure 13:**
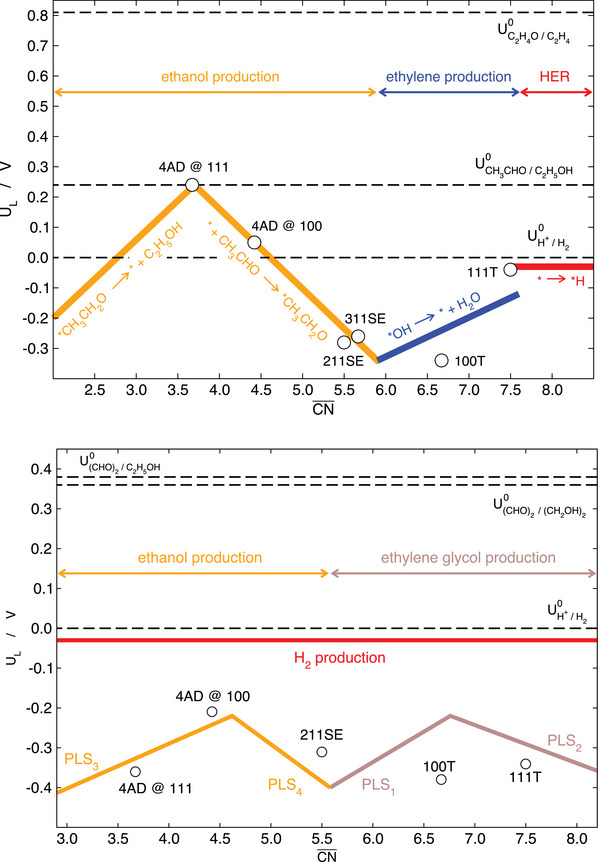
Selectivity map for some electrocatalytic reactions on Cu electrodes. Top: late stages of CO_2_ and CO reduction to C_2_ products, which correspond to ethanol production from acetaldehyde reduction versus ethylene production from ethylene oxide reduction. Bottom: ethanol versus ethylene glycol production as a result of glyoxal reduction on Cu electrodes. The dashed lines correspond to the equilibrium potentials of the competing reactions. SE: step edge, T: terrace. PLS_1_: *CHOHCHO → *CHOHCHOH, PLS_2_: *CHOHCHOH → *CHOHCH_2_OH, PLS_3_: *CH_2_CHO → *CH_3_CHO or *CH_2_CHO → CH_3_CHO, PLS_4_: *CH_3_CHO → *CH_3_CH_2_O or CH_3_CHO → *CH_3_CH_2_O. Reproduced with permission.^[^ ^74^ ^]^ Copyright 2021, Wiley. Reproduced with permission.^[^ ^75^ ^]^ Copyright 2015, Royal Society of Chemistry.

This is further exemplified in **Figure** **13** for reactions connected to the reduction of oxidized carbon species on Cu electrodes. Before analyzing the figure, it is worth mentioning that the reduction of carbon oxides (CORR), namely CO_2_ and CO, on Cu electrodes has been the subject of extensive computational and experimental research for years, particularly in the past three lustra.^[^ ^127^, ^128^, ^129^, ^130^, ^131^ ^]^ Among the most desirable products of CORR are ethylene and ethanol, which supposedly form in a common pathway starting from CO—CO coupling on Cu.^[^ ^128^, ^132^, ^133^, ^134^ ^]^ Indeed, the structural sensitivity for the adsorption of CO monomers and dimers is radically different on Cu sites^[^ ^64^ ^]^ and determines the C_1_ versus C_2_ product selectivity, while the selectivity‐determining intermediate among C_2_ species is presumably *CH_2_CHO.^[^ ^133^ ^]^ If the carbon atom of the —CHO moiety is hydrogenated, the pathway ultimately leads to C_2_H_4_. Conversely, if the —CH_2_ moiety is hydrogenated, the pathway leads to C_2_H_5_OH. In this order of ideas, the late stages of CORR resemble either oxirane reduction (CH_2_CH_2_O + 2H^+^ + 2e^−^ → C_2_H_4_ + H_2_O) when ethylene is formed, or acetaldehyde reduction (CH_3_CHO + 2H^+^ + 2e^−^ → C_2_H_5_OH) when ethanol is formed. Hence, by evaluating the structural sensitivity of those two‐electron processes, it should be possible to draw some conclusions about the mechanistic intricacies of the CORR.

The selectivity map in Figure 13 (top panel) shows that acetaldehyde reduction and oxirane reduction have dissimilar structural sensitivity:^[^ ^74^, ^135^ ^]^ the former prefers undercoordinated sites, while the latter prefers sites of intermediate coordination, particularly Cu(100). Again, at highly coordinated sites, only the HER may proceed. These conclusions were corroborated by experiments^[^ ^74^, ^135^ ^]^ and are in line with CORR results.^[^ ^136^, ^137^ ^]^

Now, the bottom panel of Figure 13 deals with the early stages of CORR on Cu electrodes. It is important to note that the coupling of two *CHO moieties under vacuum conditions was shown to be less energetically demanding than that of two *CO moieties.^[^ ^138^ ^]^ In addition, *CHOCHO was found by DFT to be an intermediate of CORR to ethanol in a pathway in which acetaldehyde is experimentally detected.^[^ ^139^ ^]^ These two pieces of information suggest that the reduction of glyoxal, (CHO)_2_, might provide important information on the first steps of CORR on Cu electrodes.

Electrochemical experiments indicated that glyoxal reduction does lead to ethanol, but important amounts of ethylene glycol are also formed, which is in contrast with CORR experiments in which ethylene glycol is only a minor product.^[^ ^75^, ^134^ ^]^ Since ethylene glycol is known to be electrochemically irreducible under regular CORR conditions,^[^ ^134^ ^]^ glyoxal is unlikely to be a major intermediate of CORR. The structural sensitivity of glyoxal reduction can be seen in the bottom panel of Figure 13. Ethanol formation is enhanced at undercoordinated sites ($\bar{\mathrm{CN}}=4.6$), while ethylene glycol formation is enhanced at sites of intermediate coordination ($\bar{\mathrm{CN}}=6.8$), which provides clear guidelines for the design of active and selective electrochemical routes toward ethylene glycol.

In perspective, Figure 13 suggests that the geometric structure of the electrodes is a major factor to modulate the selectivity of CORR and akin reactions. Of course, there are several other factors to bear in mind for these and numerous other reactions, such as (local) pH, anion/cation effects, mass transport limitations,^[^ ^127^, ^128^, ^129^, ^130^, ^131^, ^140^, ^141^, ^142^ ^]^ adsorbate coverage and spectator effects,^[^ ^143^, ^144^ ^]^ and kinetic effects.^[^ ^129^, ^145^, ^146^ ^]^ A holistic computational design of electrocatalysts should incorporate as many of those effects as possible while balancing accuracy, simplicity, and computational expenses.

## 3 Conclusions and Outlook

Generalized coordination numbers are an inexpensive and accurate descriptor for transition metals. They capture trends in adsorption energies on extended surfaces and nanoparticles altogether and can be used to elaborate electrocatalytic activity plots and selectivity maps. While they were initially defined for fully relaxed, pure metals, more recent works enabled them to incorporate strain and alloying effects.

Because they incorporate first and second nearest neighbors into the count, they tend to be more descriptive than conventional coordination numbers. Furthermore, generalized coordination numbers are analytically connected to the d‐band center, which is the archetypal descriptor for adsorption‐energy trends on transition metals.

It is worth highlighting that generalized coordination numbers outline the geometric configuration of the active sites, which is not possible by using energetic descriptors. Quantitative coordination guidelines enable an atom‐by‐atom design of active sites, as opposed to materials selection via brute‐force screening.

So far, generalized coordination numbers have primarily been used for reactions within the water and carbon cycles, and I anticipate that they may also be helpful for reactions within the nitrogen cycle. Finally, I hope that in the years to come they keep developing so as to cover new classes of materials and find new and exciting applications in electrocatalysis and other branches of catalysis.

## Conflict of Interest

The author declares no conflict of interest.
